# A Frequency Domain-Enhanced Transformer for Nighttime Object Detection

**DOI:** 10.3390/s25123673

**Published:** 2025-06-12

**Authors:** Yaru Li, Li Shen

**Affiliations:** 1 Haide College, Ocean University of China, Qingdao 266100, China; liyaru@stu.ouc.edu.cn; 2 School of Information and Electronics, Beijing Institute of Technology, Beijing 100081, China

**Keywords:** object detection, nighttime perception, frequency domain, window cross-attention fusion, mathematical statistics

## Abstract

Nighttime object detection poses significant challenges due to low illumination, noise, and reduced contrast, which can severely impact the performance of standard detection models. In this paper, we present NF-DETR (Night-Frequency Detection Transformer), a novel framework that leverages frequency domain information to enhance object detection in challenging nighttime environments. Our approach integrates physics-prior enhancement to improve the visibility of objects in low-light conditions, frequency domain feature extraction to capture structural information potentially lost in the spatial domain, and window cross-attention fusion that efficiently combines complementary features while reducing computational complexity, significantly improving detection performance without increasing the parameter count. Extensive experiments on two challenging nighttime detection benchmarks, BDD100K-Night and City-Night3K, demonstrate the effectiveness of our approach. Compared to strong baselines such as YOLOv8-M, YOLOv12-X, and RT-DETRv2-50, NF-DETR-L achieves improvements of up to +3.5% AP@50 and +3.7% AP@50:95 on BDD100K-Night, and +2.7% AP@50 and +1.9% AP@50:95 on City-Night3K, while maintaining competitive inference speeds. Ablation studies confirm that each proposed component contributes positively to detection performance, with their combination yielding the best results. NF-DETR offers a more robust solution for nighttime perception systems in autonomous driving and surveillance applications, effectively addressing the unique challenges of low-light object detection.

## 1. Introduction

Object detection in nighttime environments is a critical task for numerous computer vision applications, including autonomous driving, surveillance systems, and intelligent transportation systems. Unlike daytime scenarios, nighttime conditions present unique challenges due to low illumination, increased noise, reduced contrast, and uneven lighting distributions. These challenges can severely degrade the performance of state-of-the-art object detection models that are primarily designed and optimized for well-lit environments.

Existing literature has proposed various nighttime object detection techniques [1,2,3], which commonly model prominent vehicle light features in nighttime scenes. However, due to complex road environments, these features often suffer from occlusion or blurring, making the integration of multi-scale features and global semantic information a critical strategy for addressing these challenges.

In their research, Kuang et al. [4], Chen et al. [5], and Kuang et al. [6] applied bionic mechanism-based nighttime image enhancement methods to feature fusion and object classification. Their subsequent work combined traditional image segmentation with deep features to generate regions of interest (ROIs) for candidate box extraction. While following the multi-level feature fusion paradigm, these approaches are not end-to-end structures, resulting in complex training processes. Using CycleGAN style transfer, Ostankovich et al. [7] enhanced nighttime perception modules, but the generated images often contain visual artifacts that may interfere with object detection performance. Shao et al. [8] introduced a cascade detection network FteGanOd, comprising a Feature Translation Enhancement module (FTE) for feature translation and an object detection module for final detection. This method, however, exhibits significant missed detections for small objects at long distances and in high-speed scenarios, creating potential safety concerns.

Recent advancements in transformer-based object detection models have demonstrated remarkable performance in various detection tasks, with architectures like DETR and RT-DETR achieving state-of-the-art results. However, these models still face substantial performance degradation in nighttime scenarios primarily because they rely heavily on spatial domain features that become unreliable in low-light conditions. The spatial features in nighttime images often contain increased noise, blurred boundaries, and diminished texture details that make object localization and classification challenging.

In this paper, we propose NF-DETR (Night-Frequency Detection Transformer), a novel object detection framework specifically designed to address the challenges of nighttime perception. Our key insight is that frequency domain representations can provide complementary structural information that remains more stable under varying lighting conditions. While spatial domain features may be heavily influenced by illumination changes, frequency domain characteristics can better preserve structural patterns that are crucial for object recognition in low-light environments.

NF-DETR leverages a dual-pathway architecture that processes both spatial and frequency domain information. The spatial pathway follows a conventional object detection pipeline, while the frequency pathway extracts and processes frequency domain features using Fast Fourier Transform (FFT). These complementary features are then fused through an efficient window cross-attention mechanism that focuses on local regions where lighting conditions are more consistent, enabling a more effective integration of multi-domain information while reducing computational complexity.

**Figure 1:**
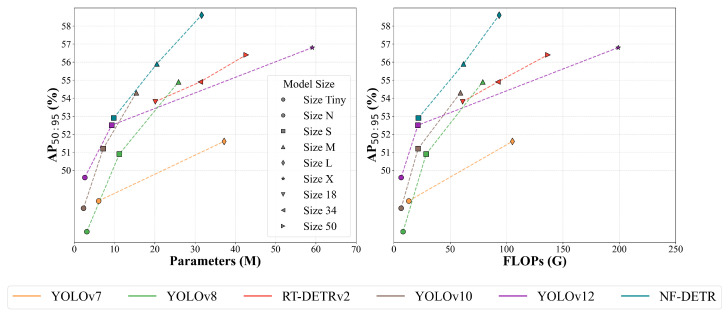
Comparison of NF-DETR with representative models on the BDD100K-Night dataset. The (**left**) panel shows AP_50:95_ (average precision from IoU 0.5 to 0.95) versus model parameters (in millions), and the (**right**) panel shows AP_50:95_ versus computational cost (FLOPs in Giga operations). Higher AP and lower parameter/FLOPs indicate better efficiency and accuracy trade-offs.

Our contributions can be summarized as follows:

1. We introduce **City-Night3K**, a new large-scale dataset specifically designed for nighttime object detection, containing 3000 diverse night scenes with fine-grained annotations across various urban environments, lighting conditions, and weather scenarios.
2. We propose innovative techniques including a **physics-prior enhancement module** that improves visibility in low-light conditions, a **frequency domain feature extraction pathway** that captures structural information potentially lost in the spatial domain, and an **efficient window cross-attention fusion mechanism** that reduces computational complexity while maintaining detection accuracy.
3. We present **NF-DETR** as a complete framework that significantly outperforms state-of-the-art methods on challenging nighttime detection benchmarks, with extensive experimental results demonstrating its effectiveness across different object categories and lighting conditions. The performance of NF-DETR on the nighttime dataset is illustrated in Figure 1.

Through comprehensive ablation studies, we verify the effectiveness of each component in our proposed framework and provide insights into how frequency domain information can complement spatial features in low-light conditions. Our experimental results show that NF-DETR achieves superior performance across different object categories and lighting conditions, making it a robust solution for nighttime perception systems.

## 2. Related Works

### 2.1. Object Detection in Nighttime Images

Recent efforts also extend nighttime detection to non-urban domains such as field robotics and agriculture. For example, Wang et al. [9] proposed a geometry-aware 3D learning framework for identifying precise cutting points under unstructured low-light conditions, while Wu et al. [10] utilized an enhanced CycleGAN to improve fruit detection robustness during nighttime harvesting scenarios.

Object detection in nocturnal environments continues to pose significant challenges due to insufficient illumination, low contrast, and the intricate mix of artificial lighting with dark backgrounds. Early efforts often centered on utilizing vehicle lights as dominant detection cues. For example, O’Malley et al. [11] applied HSV color-based segmentation to highlight headlamp regions, whereas He et al. [12] adopted decision tree classifiers relying on visual appearance features. Although these methods perform reasonably under constrained settings, they encounter difficulties when faced with occlusions or poorly lit objects.

Later, the development of traditional machine learning techniques led to broader strategies involving region proposals from intensity thresholds [13,14], saliency detection [15], and engineered descriptors [16]. Kuang et al. [4,6] enhanced low-light proposal quality by combining SVMs, edge-box region generators, and locally weighted feature selection. More recently, the research community has expanded its focus beyond vehicle detection to encompass a wider variety of nighttime traffic participants. Qin Yang et al. [17] presented a serial detection pipeline consisting of two separate modules—one for localization and one for classification—to alleviate multi-tasking stress. Their architecture incorporates lightweight depthwise separable convolutions for streamlined feature extraction and leverages attention-enhanced residual modules to improve classification. This separation of tasks provides a viable design for dealing with the complexities of night scenarios.

Meanwhile, generative adversarial networks (GANs) [18,19,20,21] have gained popularity for tackling data scarcity issues, offering synthetic training samples to bolster model performance. Shao et al. [8] further addressed visual ambiguity by developing a multi-scale enhancement scheme that suppresses interference from ambient lighting and improves object-background differentiation.

However, despite these improvements, many methods still overly depend on light-emitting features and struggle with motion blur, occlusions, and heterogeneous lighting. Additionally, frequency domain representations—potentially valuable under such adverse conditions—remain largely unexploited in existing detection models.

### 2.2. Real-Time End-to-End Object Detection

The landscape of real-time object detection has experienced rapid evolution, propelled by the emergence of highly efficient one-stage models that prioritize both speed and accuracy. Among these, the YOLO (You Only Look Once) series has maintained a central role, continuously redefining the benchmarks of detection performance through successive generational enhancements. The introduction of YOLOv3 [22] brought forth a multi-scale prediction architecture capable of handling objects at diverse scales. Building on this foundation, YOLOv4 [23] enhanced network capacity and learning ability through the incorporation of Cross Stage Partial Networks (CSPNets) and deep feature aggregation strategies. YOLOv5 [24] focused on practical deployment optimizations by implementing automated anchor box learning and pruning techniques to reduce redundancy. In later iterations, models such as YOLOv6 [25] and YOLOv7 [26] further improved performance through innovative modules like BiC, SimCSPSPPF, and E-ELAN, which facilitate efficient gradient transmission and feature reuse. The evolution continued with YOLOv8 [27], which introduced the C2f design to enhance feature compactness and representational richness. More recent versions—YOLOv9 to YOLOv12 [28,29,30,31]—have introduced groundbreaking concepts including the GELAN framework, programmable gradient flows, training paradigms without non-maximum suppression (NMS), and attention-based multi-branch fusion, collectively setting new standards in real-time detection architectures.

In addition to these CNN-based innovations, transformer-based frameworks have also emerged as promising candidates for real-time detection tasks. For instance, RT-DETR [32] brought the power of attention mechanisms and uncertainty-aware query sampling into low-latency detection scenarios. Its successor, RT-DETRv2 [33], refined this direction further by adopting enhanced encoder designs and diversified training strategies, which significantly improved adaptability and detection robustness across scenes.

Nevertheless, a critical bottleneck remains unresolved: mainstream real-time detectors are predominantly trained on daytime datasets with adequate lighting conditions, which limits their effectiveness in complex low-light environments. In such nighttime scenes, where visual degradation due to darkness, glare, or noise is prevalent, conventional models struggle to maintain their performance. Compounding this issue is their typical reliance on spatial domain features alone, thereby ignoring frequency domain cues that can offer rich complementary information in visually ambiguous settings.

To overcome these challenges, we propose a novel real-time detection framework that seamlessly integrates spatial and frequency domain representations. Specifically designed for nighttime visual perception, our method leverages frequency-aware enhancement and a multi-branch fusion mechanism to bolster the model’s resilience under adverse illumination. This unified approach not only enhances the robustness of object detection in challenging conditions but also fills a significant gap left by conventional architectures.

### 2.3. Frequency-Spatial Feature Integration

Traditional object detection frameworks primarily operate in the spatial domain, relying on pixel-level features. However, frequency domain information provides complementary advantages in modeling global structures and periodic patterns, which is especially useful under adverse conditions such as low-light and nighttime environments. Xu et al. [34] proposed a DCT-based frequency selection approach to suppress redundant components while preserving discriminative cues, enhancing both detection and segmentation tasks. In UAV-based detection, Wang et al. [35] introduced frequency domain disentanglement to improve generalization across varied scenes. CDF-Net [36] further demonstrated that fusing spatial and frequency domain features can strengthen feature representation and improve detection performance. Beyond object detection, Guo et al. [37] designed a spatial-frequency fusion network tailored for fine-grained classification on limited data, while Hong et al. [38] introduced a multi-scale feature integration strategy with Fourier transform and attention mechanisms for cross-view X-ray image classification.

Recent efforts further validate the versatility and expanding scope of frequency domain modeling across multiple vision domains. Tan et al. [39] introduce a frequency-aware learning strategy that leverages spectral domain representations to detect deepfakes more reliably, particularly under distribution shifts—demonstrating how frequency cues improve generalization in adversarial or manipulated image settings. Lian et al. [40] propose a lightweight fault diagnosis framework that integrates cross-domain image fusion and frequency-enhanced feature learning, which effectively adapts to unseen mechanical states and domains, showcasing frequency domain resilience in industrial applications. Xiang and Liang [41] explore remote sensing image compression through the explicit separation of high- and low-frequency information, emphasizing the importance of preserving structured spatial semantics during lossy compression. Collectively, these studies highlight that frequency domain features not only strengthen fine-grained spatial representations but also offer improved adaptability, robustness, and generalization under a wide range of visual degradation conditions—including those encountered in nighttime object detection.

## 3. Methodology

**Figure 2:**
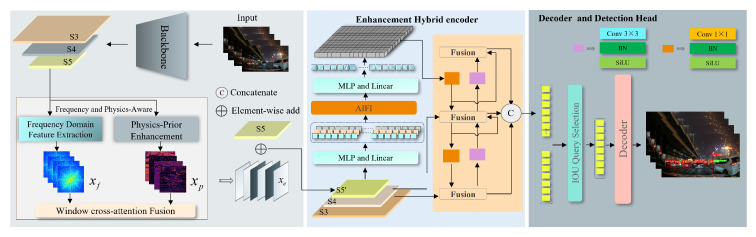
Overall architecture of the proposed real-time frequency and physics-aware dual-branch detection framework. S3, S4, and S5 denote feature maps extracted from different backbone stages; S5’ is the enhanced output. *©* represents feature concatenation; ⊕ denotes element-wise addition. MLP stands for multi-layer perceptron; $x_{f}$ and $x_{p}$ denote frequency- and physics-enhanced features, respectively. AIFI refers to Adaptive Interaction Feature Integration.

As illustrated in Figure 2, the overall architecture of our model, named NF-DETR, is as follows. A lightweight ResNet [42] backbone is used to extract multi-level semantic features from the input image, particularly from stages S3 to S5. The deep feature $X_{S5}$ is passed into our proposed Frequency and Physics-Aware Module (FPA), which consists of two parallel branches: a frequency domain enhancement branch and a physics-guided reflectance modeling branch. These two branches are designed to improve structural sensitivity and illumination robustness in low-light environments, which are essential for nighttime road object detection. The outputs from both branches are fused via a window-based cross-attention module, resulting in enhanced features $X_{e}$. These enhanced features are subsequently fed into a hybrid encoder and Transformer decoder, along with skip-connected features from stages S3 and S4, to form a rich multi-scale representation. Final predictions are produced through an IoU-aware Query Selection module followed by a detection head. Thanks to the dual-path enhancement design, window-level attention mechanism, and efficient backbone, our framework achieves high detection accuracy while maintaining real-time inference speed in challenging nighttime scenarios.

### 3.1. Frequency and Physics-Aware Module

**Figure 3:**
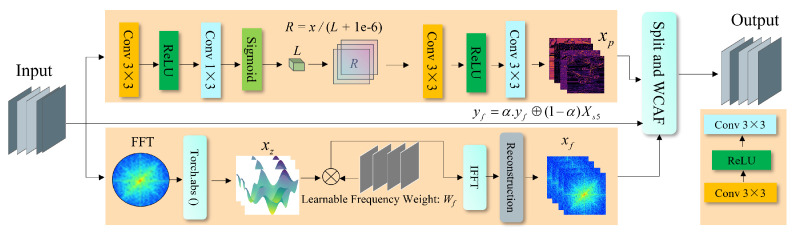
Overall architecture of the proposed Frequency and Physics-Aware Module.

The FPA module represents the core innovation of our approach for nighttime road object detection. As illustrated in the architecture diagram (Figure 3), the FPA module consists of two parallel branches: the physics-aware branch (upper path) and the frequency-aware branch (lower path), which together address the unique challenges of nighttime imagery. The motivation behind the physics-aware branch stems from the Retinex theory, which models an image as a product of illumination and reflectance. In low-light scenes, separating these components allows for enhanced visibility without over-reliance on learned priors. Compared to conventional CNN-based enhancement modules, reflectance modeling offers greater interpretability and robustness under uneven or degraded lighting. The frequency-aware branch is based on Fast Fourier Transform (FFT), which provides a global receptive field and emphasizes a high-frequency structure (e.g., edges and contours) that is often diminished in spatial domain processing. FFT is selected for its computational efficiency and simplicity over alternatives such as wavelet transform, which typically involves more parameters and requires a manual basis design. This dual-branch structure ensures that both illumination correction and structural enhancement are performed in a principled and complementary manner, making it well-suited for challenging nighttime detection tasks. The pseudocode of the FPA module is provided in Algorithm 1.
**Algorithm 1** Frequency and Physics-Aware Module (FPA).**Input:** Feature map *x* from backbone stage $S_{5}$**Output:** Enhanced feature map $y_{\mathrm{fused}}$
**1.** **Physics-Aware Branch:**
Estimate illumination using sequential convolutions: $L=\sigma ({\mathrm{Conv}}_{1\times 3}\left(\mathrm{ReLU}\left({\mathrm{Conv}}_{3\times 3}\left(x\right)\right)\right))$;Compute reflectance via Retinex normalization: R=x/(L+ϵ), where $\epsilon =10^{-6}$;Refine normalized reflectance: $x_{p}={\mathrm{Conv}}_{3\times 3}\left(\mathrm{ReLU}\left({\mathrm{Conv}}_{3\times 3}\left(R\right)\right)\right)$.
**2.** **Frequency-Aware Branch:**
Apply 2D FFT: X=F(x);Subband decomposition: $X_{s}=\mathrm{TS}\left(X\right)$;Apply frequency weights: $Y=X_{s}⊙W_{f}$;Inverse FFT: $y_{f}=F^{-1}\left(Y\right)$;(Optional) Convolutional refinement: $y_{f}={\mathrm{Conv}}_{3\times 3}\left(\mathrm{ReLU}\left(y_{f}\right)\right)$.
**3.** **Adaptive Fusion:**
Fuse branches: $y_{\mathrm{fused}}=\alpha \cdot y_{f}+\left(1-\alpha \right)\cdot x_{p}$.



#### 3.1.1. Physics-Aware Branch

The physics-aware branch explicitly models the light-scattering phenomenon that occurs under low-illumination conditions. In nighttime scenarios, light from sources such as streetlights and vehicle headlights interacts with atmospheric particles, leading to scattering effects that reduce visibility and contrast. We simulate this physical process via a sequence of convolutional operations. The pipeline begins with a 3×3 convolutional layer, followed by a ReLU activation, a 1×3 convolution, and a sigmoid activation. This structure allows initial features to be extracted while preserving spatial relations critical to scene understanding. The core of this branch is inspired by Retinex theory, which assumes that an observed image can be decomposed as the product of illumination and reflectance:

(1) I=L·R

where *I* denotes the observed image, *L* the illumination component, and *R* the reflectance. Since *R* captures the intrinsic properties of objects and is invariant to lighting, it is especially valuable for nighttime detection.

To estimate the reflectance, we apply the following transformation:

(2) $$R=\frac{x}{L+1e^{-6}}$$

where *x* is the input feature map, *L* is the estimated illumination component, and a small constant $1e^{-6}$ is added to prevent division by zero. This operation normalizes the input features by illumination and enhances object visibility in low-light regions.

The normalized output is further refined through another sequence of 3×3 convolution, ReLU activation, and 3×3 convolution, producing the physics-aware feature representation $x_{p}$, which encodes lighting-invariant scene structure for downstream detection.

#### 3.1.2. Frequency-Aware Branch

The frequency-aware branch complements the physics-aware branch by analyzing the input in the frequency domain. This design is motivated by the observation that critical structural cues are often better preserved in specific frequency bands, especially under low-light conditions.

The processing begins with a Fast Fourier Transform (FFT) that converts spatial domain features x(t) to the frequency domain:

(3) $$X\left(\omega \right)=F\left[x\left(t\right)\right]=\int_{-\infty }^{\infty }x\left(t\right)e^{-j\omega t}dt$$

For the discrete case, we apply the 2D Discrete Fourier Transform (DFT):

(4) $$X\left(u,v\right)=\frac{1}{MN}\sum_{m=0}^{M-1}\sum_{n=0}^{N-1}x\left(m,n\right)e^{-j2\pi \left(\frac{um}{M}+\frac{vn}{N}\right)}$$

We then apply a Total Subband (TS) decomposition to obtain a frequency-separ- ated representation:

(5) $$X_{s}=\mathrm{TS}\left(X\left(\omega \right)\right)$$

To emphasize informative components, learnable frequency weights $W_{f}$ are introduced, and element-wise multiplication is applied to obtain the weighted freque- ncy response:

(6) $$Y\left(\omega \right)=X_{s}⊙W_{f}$$

Next, the weighted representation is converted back to the spatial domain using the Inverse Fourier Transform:

(7) $$y_{f}=F^{-1}\left[Y\left(\omega \right)\right]=\frac{1}{2\pi }\int_{-\infty }^{\infty }Y\left(\omega \right)e^{j\omega t}d\omega$$

For discrete signals, we use the 2D Inverse DFT:

(8) $$y_{f}\left(m,n\right)=\sum_{u=0}^{M-1}\sum_{v=0}^{N-1}Y\left(u,v\right)e^{j2\pi \left(\frac{um}{M}+\frac{vn}{N}\right)}$$

Finally, we apply a reconstruction module consisting of convolutional and nonlinear operations to refine the frequency-aware features.

The final output $y_{f}$ retains essential structure from the frequency domain and is suitable for downstream fusion. To combine it with the spatial branch output $X_{S5}$, we apply adaptive fusion:

(9) $$y_{f}=\alpha \cdot y_{f}⊕\left(1-\alpha \right)X_{S5}$$

where α is a scalar that adjusts the balance between frequency and spatial contributions, and ⊕ denotes element-wise summation.

#### 3.1.3. Window Size Split for Fusion of Different Domain Features

**Figure 4:**
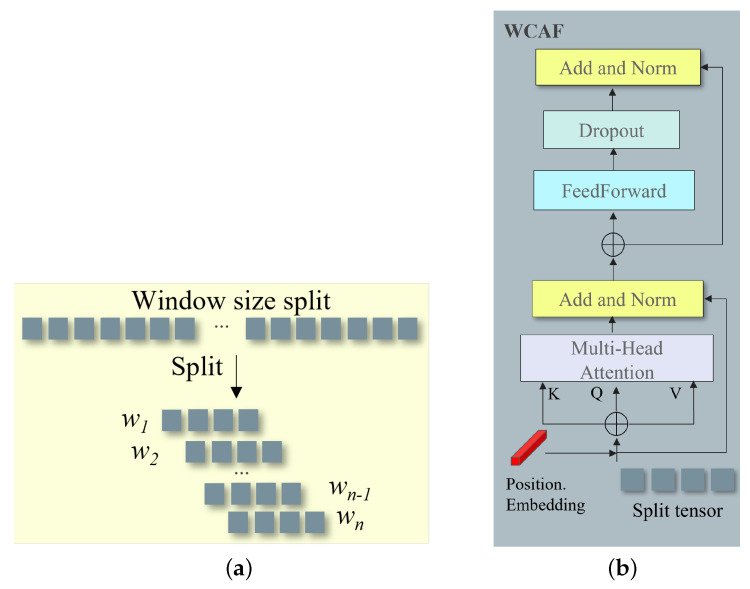
Illustration of the proposed window split and WCAF module. The window split enables local spatial grouping of features, while WCAF adaptively fuses information from both physics and frequency branches within each window. (**a**) Window-based feature splitting. (**b**) Weighted Channel Attention Fusion (WCAF) module.

To effectively integrate the complementary information from both the physics-aware and frequency-aware branches, we propose a window-based splitting approach followed by a *Weighted Channel Attention Fusion (WCAF)* mechanism. This design allows the model to attend to local spatial contexts while maintaining cross-domain feature interactions, as shown in Figure 4.

##### Window-Based Feature Splitting

The feature maps from both branches, $x_{p}$ (physics-aware) and $y_{f}$ (frequency-aware), are divided into multiple non-overlapping windows of equal size:

(10) $$F=\left\{w_{1},w_{2},\ldots ,w_{n}\right\},\;F\in \left\{x_{p},y_{f}\right\}$$

This window-based strategy serves several critical purposes:

- It reduces computational cost by operating on smaller regions.
- It enhances local context modeling, beneficial for detecting small and dim objects.
- It enables localized cross-domain feature interaction between branches.

The window size is treated as a tunable hyperparameter and selected via ablation on the target dataset.

##### Weighted Channel Attention Fusion (WCAF)

Each corresponding window pair $\left(w_{p},w_{f}\right)$ from the two domains is processed via the WCAF module. First, we concatenate each window pair to form a query tensor:

(11) $$\mathrm{query}=\mathrm{Concat}(w_{p},w_{f})$$

Positional information is embedded to preserve spatial awareness within each window:

(12) $${\mathrm{query}}^{\prime }=\mathrm{query}+\mathrm{PosEmbedding}$$

We apply a transformer-style fusion process. Specifically, we compute multi-head self-attention (MHSA) as follows:

(13) $$Q,K,V=\mathrm{Linear}({\mathrm{query}}^{\prime })$$

(14) $$\mathrm{Attention}\left(Q,K,V\right)=\mathrm{softmax}\left(\frac{QK^{⊤}}{\sqrt{d_{k}}}\right)V$$

The output is then passed through residual connections, normalization, and feed-forward layers:

(15) $$F_{1}=\mathrm{LayerNorm}\left({\mathrm{query}}^{\prime }+\mathrm{MHSA}\left({\mathrm{query}}^{\prime }\right)\right)$$

(16) $$F_{2}=\mathrm{LayerNorm}\left(F_{1}+\mathrm{FeedForward}\left(F_{1}\right)\right)$$

(17) $$F_{3}=\mathrm{Dropout}\left(F_{2}\right)$$

(18) $$F_{\mathrm{fused}}=\mathrm{LayerNorm}\left(F_{3}+\mathrm{query}\right)$$

After all window pairs are processed, the fused outputs are recombined to form the final output feature map:

(19) $$F_{\mathrm{out}}=\mathrm{Recombine}\left(\left\{F_{\mathrm{fused}}^{1},F_{\mathrm{fused}}^{2},\ldots ,F_{\mathrm{fused}}^{n}\right\}\right)$$

##### Advantages and Effectiveness

This localized fusion approach offers several advantages:

- Spatially adaptive fusion across regions with varying illumination.
- Reduced self-attention cost due to per-window computation.
- Better retention of fine-grained details from both frequency and physics domains.

The resulting feature map $F_{\mathrm{out}}$ preserves complementary strengths of both domains—enhanced visibility via illumination modeling and structural fidelity via frequency analysis—and is passed into subsequent detection heads for final object detection.

### 3.2. Decoder and Detection Head

In our proposed framework, the decoder and detection head play a crucial role in processing the enhanced features from the FPA module. The architecture adopts a transformer-based structure with specific optimizations for nighttime road object detection.

The decoder receives the fused feature representation $F_{\mathrm{out}}$ and processes it through *L* sequential transformer decoder layers:

(20) $$Z_{l}={\mathrm{DecoderLayer}}_{l}\left(Z_{l-1},F\right),\;l=1,2,\ldots ,L$$

Here, $F\in R^{N\times C}$ denotes the encoder output, and $Z_{0}=Q$ represents the initial learnable query embeddings, with $Q\in R^{M\times C}$. Each decoder layer performs cross-attention to align queries with relevant image regions. To better handle illumination variability, we incorporate physics-guided attention that adaptively adjusts attention weights based on estimated lighting, thereby suppressing false detections from reflective or overexposed areas.

The final decoder output $Z_{L}\in R^{M\times C}$ is passed to two parallel branches:

- A classification branch that outputs predicted class probabilities $\hat{c}\in R^{M\times K_{c}}$.
- A regression branch that predicts bounding boxes $\hat{b}\in R^{M\times 4}$.

To better address nighttime-specific challenges, we design an objective function that includes both geometric and categorical constraints:

(21) $$L\left(F,\hat{Y},Y\right)=L_{\mathrm{bbox}}\left(\hat{b},b\right)+L_{\mathrm{cls}}\left(\Phi \left(F\right),\hat{c},c\right)$$

where $\hat{Y}$ and *Y* denote the predicted and ground truth annotations, with object category c and location b. The discrepancy function Φ(·) captures the mismatch between positional and categorical distributions:

(22) Φ(F)≜|P(F)−C(F)|

This comprehensive detection head enables precise object localization and classification under nighttime driving conditions. By incorporating illumination-adaptive scoring and physics-informed attention, our system achieves reliable performance across a range of visually degraded scenarios involving glare, reflections, and shadows.

## 4. Experiment

### 4.1. Datasets

#### 4.1.1. Experimental Datasets

To evaluate the performance of our proposed framework, we conducted extensive experiments on two distinct nighttime datasets: a carefully filtered subset of BDD100K and our newly collected and annotated City-Night3K dataset.

**BDD100K-Night Subset:** We extracted a specialized nighttime subset from the Berkeley Deep Drive (BDD100K) dataset, which is one of the largest and most diverse driving video datasets available. Through timestamp-based filtering and illumination-aware analysis, we selected 28,653 nighttime images representing diverse urban and suburban driving scenes under low-light conditions. For the detection task, the original fine-grained labels were consolidated into four primary categories critical to nighttime safety: *car*, *person*, *truck*, and *rider*. This consolidation helps the model focus on safety-critical object types while maintaining robustness under challenging lighting conditions.

**City-Night3K Dataset:** To address the limitations of existing datasets in modeling real-world urban nighttime driving, we constructed a new dataset—City-Night3K—focused on challenging nighttime traffic conditions. The dataset comprises 3560 high-resolution images (2048 × 1024) collected between 19:00 and 04:00 from publicly accessible traffic surveillance cameras operated by the transportation departments of Zhejiang and Chengdu, China. These images cover a variety of urban nighttime scenes, including intersections, highways, tunnels, and rainy or low-visibility conditions.

All images were manually annotated using the LabelImg tool, with a total of 93,230 bounding boxes spanning four safety-critical object categories: car, person, truck, and rider—consistent with the taxonomy used in the BDD100K-Night subset. A rigorous two-stage quality assurance protocol was employed, including initial annotation by trained staff and subsequent verification by senior reviewers to ensure annotation precision.

**Figure 5:**
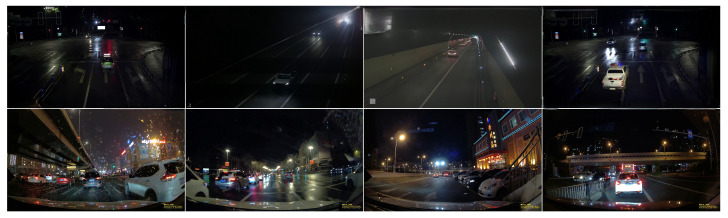
Representative samples from the City-Night3K dataset, highlighting illumination diversity, reflections, shadows, and partial visibility.

Figure 5 illustrates several sample images from the City-Night3K dataset. These samples reflect realistic visual challenges, including strong glare, deep shadows, wet surface reflections, and partially illuminated objects—common in real-world nighttime urban environments.

#### 4.1.2. Data Distribution and Characteristics

Both BDD100K-Night and City-Night3K datasets were partitioned into training, validation, and testing sets with a 70%/10%/20% split. For City-Night3K, we ensured balanced coverage across diverse nighttime conditions, including varied illumination (e.g., streetlights, headlight-only, and backlit), weather types (clear, rainy, and foggy), and traffic densities.

Beyond general diversity, both datasets also reflect key real-world detection challenges. Overlapping and occluded instances are common in scenes such as intersections, tunnel entrances, and multi-lane roads—particularly involving pedestrian groups, vehicles at close proximity, and rider–motorcycle pairs. These cases were preserved during annotation.

In addition, rare object classes were deliberately retained to reflect natural frequency imbalance. For example, in City-Night3K, categories like *truck* and *rider* appear in fewer than 15% of the annotated frames, often under adverse visual conditions. This supports the evaluation of model generalizability to underrepresented targets.

Together, these properties form a comprehensive and realistic platform for benchmarking nighttime object detection under spatial occlusion, illumination degradation, and class imbalance. They enable the reliable assessment of model robustness and transferability in real-world deployment scenarios.

### 4.2. Evaluation Metrics

To thoroughly assess our framework’s performance in nighttime object detection scenarios, we adopted a comprehensive set of metrics that evaluate both detection accuracy and computational efficiency. Detection accuracy is primarily measured using the average precision (AP) metric across varying Intersection over Union (IoU) thresholds. We report

- **mAP@50:** Mean AP at IoU threshold of 0.5.
- **mAP@50:95:** Averaged over IoUs from 0.5 to 0.95 with a 0.05 step.

These metrics offer insights into both localization precision and classification accuracy. AP is computed from the precision–recall (PR) curve, where precision is TP/(TP+FP) and recall is TP/(TP+FN). This evaluation is especially important under nighttime conditions, where models frequently face false positives due to light sources and false negatives caused by low object visibility.

In addition to accuracy, we assess computational efficiency using

- **Parameter count (M):** Total number of learnable parameters.
- **FLOPs (GFLOPs):** Giga floating-point operations.
- **Inference speed (FPS):** Frames processed per second.

These metrics determine the model’s feasibility for real-time deployment in autonomous driving and surveillance systems, where latency and memory are critical.

### 4.3. Experimental Details

We implemented our framework using PyTorch 2.1.0 and conducted training on dual NVIDIA RTX 3090Ti GPUs (Nvidia, Santa Clara, CA, USA). All models were trained with a fixed input resolution of 640×640 and a batch size of 32 for fair comparison. Optimization was performed using AdamW with

- Learning rate: $1\times 10^{-4}$ for the full model; $1\times 10^{-5}$ for the backbone [30].
- Weight decay: 0.0001, gradient clipping norm: 0.1 [30].
- EMA decay: 0.9999 [30].
- Warm-up: Linear warm-up with start factor 0.001 for 2000 steps [30].

To ensure stable training, BatchNorm layers were frozen. Our architecture included 3 RepBlocks with embedding dimension of 256, feedforward dimension of 1024, and 8 attention heads—an efficient configuration determined through ablation studies. For the frequency-spatial fusion mechanism, the scaling parameter α was initialized to 0.6 and learned during training. The window size for attention was set to 8, balancing complexity and receptive field effectiveness in nighttime environments. All experiments were conducted on Ubuntu 20.04 (64-bit) with Python 3.8, OpenCV 3.4.0, and CUDA 12.1. The detailed NF-DETR model configurations are summarized in Table 1.

### 4.4. Comparison with SOTA Methods

We evaluated the proposed NF-DETR on two challenging nighttime object detection benchmarks: BDD100K-Night and City-Night3K. For fair comparison, we benchmarked our method against several state-of-the-art detectors, including YOLOv7 [23], YOLOv8 [24], YOLOv10 [26], YOLOv12 [28], and RT-DETRv2 [30]. All models were tested with an input resolution of 640×640, and results are reported in terms of AP@50, AP@50:95, FPS, parameter count (M), and FLOPs (G). We also evaluated three variants of our method—NF-DETR-S (small), NF-DETR-M (medium), and NF-DETR-L (large)—to assess the trade-off between accuracy and efficiency.

Table 2 and Table 3 present detailed comparisons on the BDD100K-Night and City-Night3K datasets, respectively. Our NF-DETR-L achieves the highest performance on both datasets, with 82.1% AP@50 and 58.6% AP@50:95 on BDD100K-Night, outperforming RT-DETRv2-50 by 1.2% and 2.2%, respectively. Moreover, it uses 26% fewer parameters (31.6 M vs. 42.7 M), offering a better accuracy–efficiency trade-off. NF-DETR-M performs comparably to RT-DETRv2-34 while using 34.5% fewer parameters. NF-DETR-S achieves 76.3% AP@50, outperforming YOLOv10-M with a higher FPS of 111.3. On City-Night3K, NF-DETR-L again outperforms all competitors, achieving 83.6% AP@50 and 59.6% AP@50:95. Even NF-DETR-M surpasses RT-DETRv2-50 while requiring less than half the parameter size. These consistent improvements confirm the effectiveness of our proposed frequency-aware design. By enhancing structural detail preservation and fusing spatial and frequency representations through an adaptive attention mechanism, NF-DETR demonstrates strong robustness across diverse nighttime driving scenarios.

### 4.5. Ablation Study on Key Modules

To validate the effectiveness of each major component in the proposed NF-DETR framework, we conducted a series of ablation experiments on the BDD100K-Night and City-Night3K datasets. All experiments were based on the medium-scale variant (NF-DETR-M) to maintain a consistent trade-off between performance and efficiency.

We focused on the following components:

1. **Physics-Prior Image Enhancement**: improves visibility in dark environments via a decomposition-inspired illumination prior.
2. **Frequency Domain Feature Extraction**: extracts high-frequency structural cues using FFT-based transforms.
3. **Windowed Cross-Attention Fusion (WCAF)**: facilitates local spatial integration between frequency-aware and spatial features.

We defined five experimental configurations:

- **Setting 1**: Baseline DETR without any of the above components;
- **Setting 2**: Baseline + Frequency Domain Only;
- **Setting 3**: Baseline + Physics Enhancement Only;
- **Setting 4**: Baseline + Physics + Frequency;
- **Setting 5**: Full NF-DETR with Physics + Frequency + WCAF.

The results in Table 4 and Table 5 indicate that each module contributes positively to performance. Frequency-aware features yield structural robustness, physics-guided enhancement improves visibility, and WCAF effectively integrates both domains.

### 4.6. Effect of Window Size in Cross-Attention Fusion

To evaluate the impact of window size in the proposed window-based cross-attention fusion module, we conducted ablation experiments on the BDD100K-Night dataset using the NF-DETR-M variant. We tested five different window sizes: 1, 2, 4, 8, and 16. Across all settings, the model used an embedding dimension d=256, 8 attention heads, and a feedforward dimension of 1024. For settings with larger windows (4, 8, and 16), square-root scaling was applied to maintain consistent parameter counts.

As shown in Table 6, window size has a notable effect on both accuracy and efficiency. Increasing the window size from 1 to 8 yields improved detection performance, while simultaneously reducing computational cost per fusion layer—from 0.17 GFLOPs to 0.021 GFLOPs. The best performance is achieved with a window size of 8, where the model reaches 79.9% AP@50 and 55.9% AP@50:95. This improvement is attributed to the balance between capturing sufficient local context and maintaining spatial consistency within moderately sized windows, particularly important for nighttime scenes with localized illumination. When the window size increases further to 16, accuracy degrades despite the highest computational efficiency (0.011 GFLOPs). This indicates that overly coarse windows may fail to capture the relevant detail necessary for accurate detection.

**Conclusion:** A window size of 8 provides the optimal trade-off between detection accuracy and computational efficiency in nighttime object detection.

### 4.7. Ablation Study on Fusion Weight

To evaluate the impact of the fusion weight α in balancing spatial and frequency domain features, we conducted a controlled ablation study by varying α∈{0.1,0.2,0.4,0.6,0.8} while keeping all other components fixed, where ⊕ denotes element-wise addition, and α is either a fixed scalar or a learnable parameter. In this experiment, we tested static values to better isolate their influence.

**Figure 6:**
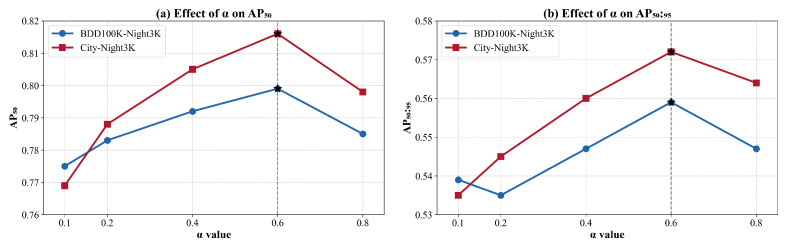
Effect of different α values on detection performance. AP_50_ refers to average precision at IoU 0.5, and AP_50:95_ is the mean AP over IoU thresholds from 0.5 to 0.95. Evaluations are conducted on both BDD100K-Night3K and City-Night3K datasets to study the impact of fusion weight α in combining spatial and frequency features. The star symbol (*) denotes the configuration adopted in the final model.

We performed the evaluation on two benchmark nighttime datasets: **BDD100K-Night3K** and **City-Night3K**, using the same backbone, training schedule, and optimization settings across all α values. Performance was assessed using both AP_50_ and AP_50:95_ metrics. As illustrated in Figure 6, we observe a consistent trend across both datasets: model performance improves as α increases from 0.1 to 0.6, then slightly drops at α=0.8. Specifically, the best performance is achieved at α=0.6, where BDD100K-Night3K achieves AP_50_ of 0.7986 and AP_50:95_ of 0.5591, while City-Night3K peaks at AP_50_ of 0.8153 and AP_50:95_ of 0.5732. This suggests that a moderate emphasis on frequency domain cues enhances detection, especially under poor illumination. However, overly increasing α diminishes the contribution of spatial priors, leading to degraded performance. These results justify the choice of setting α to 0.6 in the final model.

### 4.8. Visualization Results

**Figure 7:**
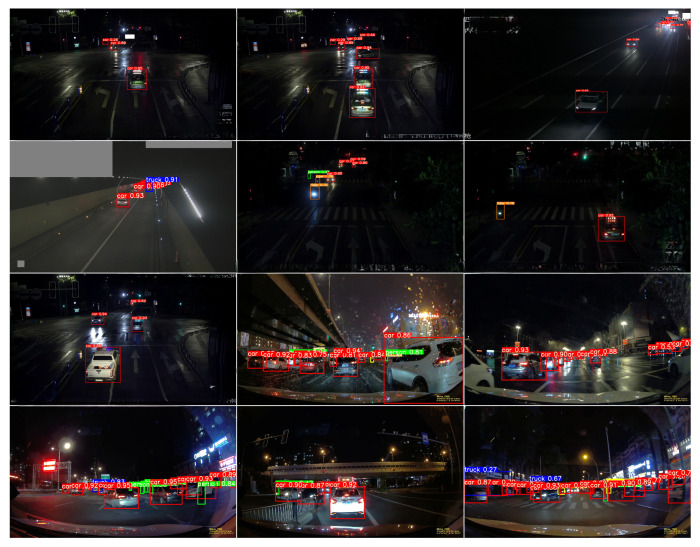
Detection results of NF-DETR-L across various nighttime urban scenes, including intersections, tunnels, and rainy highways.

To qualitatively demonstrate the capability of NF-DETR in challenging nighttime environments, we visualize detection results from diverse scenes in Figure 7. The model reliably localizes vehicles and pedestrians under tunnels, reflective roads, and rainy conditions.

**Figure 8:**
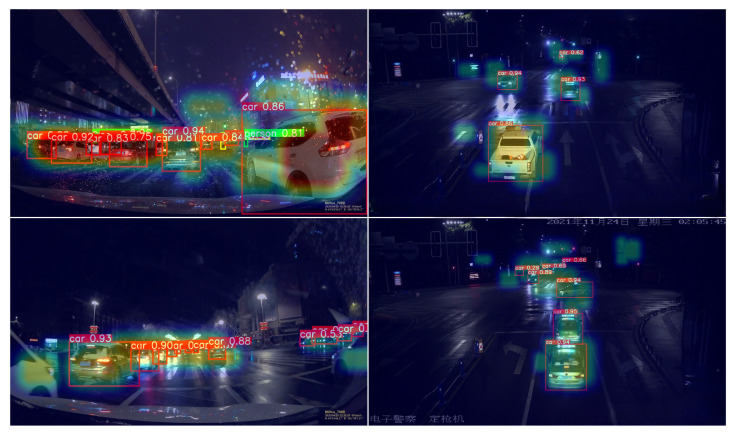
Heatmap visualizations from NF-DETR-L under various nighttime conditions. Brighter regions indicate the model’s attention focus, typically aligned with high-confidence detections such as vehicles or pedestrians. These attention maps help interpret the model’s behavior in low-light scenarios.

In addition, we provide decoder attention heatmaps in Figure 8. These visualizations indicate that NF-DETR attends to semantically meaningful regions, such as object contours and headlights, even under strong glare or occlusion.

## 5. Conclusions and Limitations

In this paper, we present **NF-DETR**, a novel dual-branch framework that leverages both frequency prior and physics-guided enhancement for robust nighttime object detection. By integrating frequency domain representations to capture stable structural patterns and incorporating physics-prior illumination modeling for contrast enhancement, NF-DETR significantly improves feature quality in low-light scenarios. Furthermore, our proposed Window Cross-Attention Fusion (WCAF) module enables effective domain-aware feature fusion with minimal computational overhead. Extensive experiments on two challenging datasets—BDD100K-Night and City-Night3K—demonstrate that NF-DETR consistently surpasses existing methods in terms of detection accuracy and real-time performance. Ablation studies further validate the independent and complementary contributions of each core module. Despite these advantages, NF-DETR still encounters challenges under extreme nighttime conditions, such as saturated glare, heavy occlusion, and severe motion blur, where even frequency cues can become unreliable. Additionally, the current frequency prior module uses fixed-window strategies and lacks adaptivity to spatial heterogeneity, potentially limiting its expressiveness in regions with high structural complexity. Although the framework remains lightweight, further optimization is required to facilitate deployment on low-power embedded platforms.

## Figures and Tables

**Table 1:** NF-DETR model configurations.

Model	Backbone	# Decoder Layers
NF-DETR-S	ResNet18	1
NF-DETR-M	ResNet34	2
NF-DETR-L	ResNet50	4

**Table 2:** Comparison of different models on the BDD100K-Night dataset.

Model	AP@50	AP@50:95	FPS	Params (M)	FLOPs (G)
YOLOv7-Tiny [23]	0.701	0.483	106.6	6.03	13.2
YOLOv7 [23]	0.743	0.516	81.4	37.2	105.2
YOLOv8-N [24]	0.676	0.466	121.9	3.1	8.2
YOLOv8-S [24]	0.736	0.509	104.6	11.2	28.7
YOLOv8-M [24]	0.786	0.549	63.5	25.9	78.9
RT-DETRv2-18 [30]	0.775	0.538	71.3	20.1	61
RT-DETRv2-34 [30]	0.790	0.549	90.4	31.3	92.3
RT-DETRv2-50 [30]	0.809	0.564	78.6	42.7	136.6
YOLOv10-N [26]	0.663	0.479	58.4	2.3	6.5
YOLOv10-S [26]	0.726	0.512	126.5	7.2	21.6
YOLOv10-M [26]	0.762	0.543	101.3	15.4	59.1
YOLOv12-N [28]	0.661	0.496	123.1	2.6	6.5
YOLOv12-S [28]	0.735	0.525	109.5	9.3	21.4
YOLOv12-X [28]	0.803	0.568	63.7	59.1	199.0
NF-DETR-S	0.763	0.529	**111.3**	9.8	21.8
NF-DETR-M	0.799	0.559	86.7	20.5	61.8
NF-DETR-L	**0.821**	**0.586**	81.7	31.6	93.4

**Table 3:** Comparison of different models on the City-Night3K dataset.

Model	AP@50	AP@50:95	FPS	Params (M)	FLOPs (G)
YOLOv7-Tiny [23]	0.726	0.493	106.6	6.03	13.2
YOLOv7 [23]	0.769	0.543	81.4	37.2	105.2
YOLOv8-N [24]	0.701	0.496	121.9	3.1	8.2
YOLOv8-S [24]	0.759	0.526	104.6	11.2	28.7
YOLOv8-M [24]	0.809	0.564	63.5	25.9	78.9
RT-DETRv2-18 [30]	0.794	0.556	71.3	20.1	61
RT-DETRv2-34 [30]	0.803	0.564	90.4	31.3	92.3
RT-DETRv2-50 [30]	0.816	0.577	78.6	42.7	136.6
YOLOv10-N [26]	0.684	0.493	58.4	2.3	6.5
YOLOv10-S [26]	0.747	0.536	126.5	7.2	21.6
YOLOv10-M [26]	0.783	0.559	101.3	15.4	59.1
YOLOv12-N [28]	0.691	0.513	123.1	2.6	6.5
YOLOv12-S [28]	0.756	0.551	109.5	9.3	21.4
YOLOv12-X [28]	0.803	0.584	63.7	59.1	199.0
NF-DETR-S	0.786	0.553	**111.3**	9.8	21.8
NF-DETR-M	0.816	0.572	86.7	20.5	61.8
NF-DETR-L	**0.836**	**0.596**	81.7	31.6	93.4

**Table 4:** Ablation results on City-Night3K of key modules. A checkmark ✓ indicates that the corresponding module is included in the setting. Bold values highlight the best performance in each metric.

Setting	Phys.	Freq.	WCAF	AP@50	AP@50:95
1				0.801	0.561
2		✓		0.806	0.563
3	✓			0.804	0.562
4	✓	✓		0.811	0.565
5	✓	✓	✓	**0.816**	**0.572**

**Table 5:** Ablation results on BDD100K-Night of key modules. A checkmark ✓ indicates that the corresponding module is included in the setting. Bold values highlight the best performance in each metric.

Setting	Phys.	Freq.	WCAF	AP@50	AP@50:95
1				0.779	0.542
2		✓		0.783	0.546
3	✓			0.786	0.551
4	✓	✓		0.789	0.553
5	✓	✓	✓	**0.799**	**0.559**

**Table 6:** Effect of window size on BDD100K-Night dataset.

Window Size	Params (M)	GFLOPs	AP@50	AP@50:95
1	0.79	0.170	0.796	0.554
2	0.79	0.084	0.791	0.551
4	0.79	0.042	0.794	0.553
8	0.79	0.021	**0.799**	**0.559**
16	0.79	0.011	0.776	0.546

## Data Availability

The data presented in this study are available on request from the corresponding author.
